# Use of Artificial Intelligence for Analyzing Kidney Stone Composition: Are We There Yet?

**DOI:** 10.1016/j.mcpdig.2023.06.007

**Published:** 2023-07-28

**Authors:** Shahin Isha, Sadia Z. Shah

**Affiliations:** Department of Critical Care, Mayo Clinic, Jacksonville, FL; Division of Lung Failure and Lung Transplant, Department of Transplant, Mayo Clinic, Jacksonville, FL

Kidney stones are associated with significant morbidity and are linked to over $5 billion in economic burden annually, secondary to treatment costs, and lost worker productivity.^1^^,^^2^ Unfortunately, the prevalence of kidney stones has been increasing annually over the years.^3^ Therefore, efforts to study the use of Artificial Intelligence (AI) to assist with the diagnosis, treatment, and prevention of kidney stones have intensified over the last 2 decades.^4^^,^^5^ Because it is crucial to accurately diagnose kidney stones for patients to get appropriate treatment and prevention recommendations, Day et al^6^ used AI for quality assurance during kidney stone spectra analysis to minimize erroneously classified spectra by clinical laboratory technologists.

The role of AI for kidney stone management has matured considerably since 2000, with 14 studies performed to assess diagnostic prediction, 10 to assess imaging, 9 to assess stone composition, 3 studies to assess procedure outcomes, 12 to assess extracorporeal shockwave lithotripsy, 4 to assess percutaneous nephrolithotomy, and 3 studies to assess ureteroscopy.^5^ Accurate analysis of stone composition is the most crucial diagnostic procedure to ensure that the patient is getting appropriate treatment and prevention against the recurrence of kidney stones. Common techniques used for kidney stone composition analysis are infrared spectroscopy, X-ray diffraction, and chemical analysis. In 2016, a multicenter study was performed to assess the quality of urinary stone analysis in laboratories within Europe.^7^ This study highlighted the fact that only 56% of the laboratories met the quality requirements to accurately analyze the urinary stones across 8 countries in Europe. The study also emphasized the importance of appropriate equipment, reference spectra, and the qualification of the laboratory staff technologists for an accurate analysis.

This lack of accuracy in half the laboratories has inspired the need to assess the role of AI in improving the quality of kidney stone composition analysis. There have been several studies performed so far to assess the role of AI in detecting stone composition.^8^, ^9^, ^10^, ^11^, ^12^, ^13^, ^14^, ^15^ These studies have been summarized in the Table. All these studies were able to successfully assess the composition of the kidney stones with over 90% accuracy. What is remarkable is that they were able to do that despite the smaller sample sizes used for machine learning and deep learning in the majority of these studies. With these results, it can be inferred that larger sample sizes can further improve the accuracy of AI applications.

**Table:** Studies Assessing the Role of AI in the Detection of Stone Composition

Reference, year	Sample Size (n)	Technique Used	Outcome of Interest	Accuracy
Bejan et al,^8^ 2019	Mined data from >125 million notes in EHR and identified 11,585 patients	StoneX, a natural language processing (NPL) algorithm	Non-local means algorithm performance in detecting stone composition from EHR	Positive predictive values: uric acid stones: 87.5%. Rest of the stones: >90%
Kazemi and Mirroshandel,^9^ 2018	936 patients with nephrolithiasis	Data were preprocessed to improve model performance and various data mining models, such as Bayesian model, decision trees, artificial NNs, and rule-based classifiers were employed. The final model was an ensemble learning model combining individual classifiers	Predict the chances of nephrolithiasis and kidney stone type based on various risk factors	The final ensemble-based model showed a 97.1% accuracy in predicting kidney stone type
Aldoukhi et al,^10^ 2019	63 human kidney stones of various composition	Deep convolutional neural network (DCNN) model based on digital photographs of stones	Network prediction recall in identifying kidney stone composition from digital photographs	Composition prediction recall for uric acid: 94%, calcium oxalate: 90%, cysteine: 75%, triple phosphate: 86%. Overall weighted recall: 85%
Kreigshauser et al,^11^ 2016	32-stone dataset	Single source Dual Energy Computerized Tomography (ssDECT) scan-based multiparametric model comprising Artificial Neural Network (ANN), Support Vector Machine (SVM), Decision Tree (C4.5), RandomTree, and Naïve Bayes Tree (NBTree) algorithms	Distinguishing uric acid from non-uric acid stone. Distinguishing subtypes of non-uric acid stone	For stones >5 mm: 100% accuracy in distinguishing uric acid from non-uric acid stones. 75% accuracy in non-uric acid stone subtype diagnosis
Große Hokamp et al,^12^ 2020	200 kidney stones	Dual-energy CT images were used to train ML and shallow NN	Evaluate whether ML using shallow NN enables the prediction of a stone’s main component-base and if there is dose-independent diagnostic accuracy obtained with the NN	Nearly 90% accuracy was achieved in predicting the main stone composition for stone sizes 3-18 mm
Saçlı et al,^13^ 2019	105 naturally occurring renal calculi samples collected from 40 different patients	Microwave dielectric properties of various types of renal calculi were fitted with Cole-Cole parameters and the k-nearest neighbor ML algorithm was used	Evaluated whether the ML algorithm can accurately classify renal calculi compositions	98.2% accuracy was achieved for renal calculi classification using Cole-Cole parameters
Cui et al,^14^ 2021	98 patients with infective stones and 59 with noninfective	Radiomics features extracted from CT images were processed by the LASSO algorithm, then ensemble learning based on bagged trees was used	Performance of the model in differentiating noninfection vs infection kidney stone	90.7% accuracy was achieved in differentiating noninfective vs infective kidney stones
Zhang et al,^15^ 2018	18 urate stones and 32 nonurate stones from 45 patients	CT texture analysis (CTTA)-based features were used to train SVM classifiers	Diagnostic accuracy in differentiation uric acid stone from non-uric acid stone based on unenhanced CT images	Average SVM accuracy for differentiating stone subtypes varied from 88 to 92% (after 10-fold cross-validation)

CT, computerized tomography; EHR, electronic health record; ML, machine learning; NN, neural network.

On the contrary, Bejan et al^8^ used a natural language processing-based pattern-matching algorithm that was developed to extract kidney stone composition-related information from a database comprising 125 million clinical notes. On the basis of more than 45,235 matching texts from the clinical notes of 11,585 patients, the algorithm achieved a positive predictive value of >90% in identifying the most common stone-type-related information. The algorithm also enabled them to match this information with 14 patient phenotypes related to International Classification of Diseases, Nineth Revision diagnoses (comorbidities and other clinical characteristics) against 6 different stone types, and they were able to establish associations among them. For example, in their adjusted multivariate regression-based analysis, uric acid stones were found to be associated with type 2 diabetes mellitus (odds ratio [OR], 2.69; 95% confidence intervals [CI], 1.91-3.79), often reported in the literature, and hydroxyapatite stones were associated with pulmonary collapse (OR, 3.67; 95% CI, 2.10-6.42), not reported previously. Moreover, a survival analysis from a second stone surgery in their cohort showed statistically significant differences among different stone types (*P*=.03).^8^ These findings allude to the notion that AI holds great promise for its role in diagnosing kidney stones in the future.

The strength of study reported by Day et al^6^ stems from their access to a large institutional historical database, which was developed by clinically validated Fourier transform infrared spectroscopy (FTIR)—assisted analysis of >1,000,000 kidney stones over several years (The Metals Laboratory at Mayo Clinic). This database, in conjunction with an internally developed FTIR spectra library with >300 different stone composition spectra, laid a strong foundation for the development, training, and validation of their machine-learning algorithm. Although spectra readouts from FTIR instruments and technologist-derived interpretations of the spectra were used in phase 1 (algorithm development) of their study, in a prospective phase 2, they also developed a quality assurance initiative using this program, wherein a report was generated twice a week to look at the discordance rates between AI program interpretation and human interpretation (as documented by the technologists in the Laboratory Information System). Incongruent interpretations were then reviewed by an independent qualified technologist to determine the final interpretation, and the rate of verified human misclassifications resulting in a revised report was calculated as a primary outcome. Over the 1-year study period, 81,517 kidney stone spectra were reviewed, and the overall revision rate of misreported kidney stone spectra was nearly 8 times higher (relative risk, 7.9; 95% CI, 4.1-15.2) when compared to the year before initiating the AI quality assurance program. However, it is worth noting that even though the misreported spectra identified by AI were 7.61 per 10,000 spectra during the study period, the overall event rate was still very low at 0.096% (78 of the 81,517).^6^

The role of explainable AI (XAI) in understanding the reasoning and mechanism behind model-based predictions has been well-established in Clinical Decision-Support Systems.^16^ A study by Day et. al^6^ determined the need to explore the relationship between the convolutional neural network and its associated predictions, as shown by their model, through the incorporation of SHapley Additive exPlanation (SHAP) plots using 600 training samples as the background, across a range of correct and incorrect predictions. Their SHAP prediction model looked at heatmap intensities, which represented the relative weights of spectra locations contributing to the model predictions, and areas of increased heatmap intensity corresponding to unique wavelength regions for specific stones. With the help of qualified technologists, such areas of increased heatmap intensity were reviewed and confirmed to represent specific wavelength regions used to identify other commonly encountered kidney stones. Clearly, the incorporation of a multidisciplinary clinical team including laboratory staff, data scientists, and biostatisticians, along with collaborators in clinical practice, gave further strength to their study in that it was not being performed in silos using just a database but was being tried and tested in the real world.^6^

Because 60-70% of all diagnoses are based on laboratory data, a laboratory error, even if it is happening at a low rate, can considerably affect the care of patients on a national level and can add to the economic burden on the society. The COVID-19 pandemic has highlighted, how much our health care is dependent on its workforce to ensure patient care delivery. Human-AI-augmented workflows can potentially reduce the workload on the staff in health care by providing assistance in repetitive task execution and minimizing staff burnout. With the increased use of digital information, AI-based computation pathology modalities have emerged as promising tools for increasing the accuracy and availability of high-quality care delivered to patients.^17^ However, with the development of more such machine-learning-based models, there might be a noticeable difference in their performance. Therefore, there remains a need to compare the performance of such models created for similar purposes using standard metrics. Sensitivity, specificity, positive predictive value, negative predictive value, accuracy, precision, recall, confusion matrix, receiver operating characteristics, the area under the curve, and F1 score are some of the commonly used tools to assess such models.

The Table summarizes the performance of metrics of some of the models relevant to this paper. To achieve better model performance, the rigor and characteristics of the training dataset play a great role. Although the robustness of such models is expected to improve with bigger and more diverse datasets, it may not always be feasible.^18^^,^^19^ In order to overcome such limitations, several strategies are often incorporated into model development and training. Sharing data from different sites, which vary in population and disease characteristics, often allows models to be tested and fine-tuned to perform well in varied situations. However, with ethical, legal, and logistical barriers associated with it, it often becomes challenging. Data augmentation is another approach wherein synthetic data is generated using the characteristics of the available dataset, often preserving the labels of the original dataset.^20^^,^^21^ On the contrary, transfer learning uses the existing solution for a similar problem as a starting point and tries to solve a new classification problem, thus requiring less training data to obtain a robust solution. Therefore, data sharing, data augmentation, and transfer learning modalities confer added strength to the existing machine-learning modalities by reducing the possible bias from the training dataset, optimizing the models for the new, unseen data, and increasing generalizability.^22^

Human-AI-driven management strategies hold great promise for the future and provide a roadmap for providing more personalized patient care. Because of AI being in its nascent phase, some challenges still exist, with most hospitals not having the information technology infrastructure needed and the ability of scientific societies to review and validate these technologies as they are developed and assess their role in management guidelines. Lack of insurance coverage for human-AI augmented workflows may also pose a challenge for the implementation of these applications in clinical practice if not addressed by scientific societies. A shift in clinical paradigm is forthcoming with AI algorithms taking their place in the management guidelines for kidney stone disease, but we are not there yet.

## Potential Competing Interests

The authors report no competing interests.
